# Self-forgiveness is associated with increased volumes of fusiform gyrus in healthy individuals

**DOI:** 10.1038/s41598-023-32731-0

**Published:** 2023-04-04

**Authors:** Hyun-Ju Kim, Junghwa Seo, Minji Bang, Sang-Hyuk Lee

**Affiliations:** 1 Department of Psychiatry, CHA Bundang Medical Center, CHA University School of Medicine, 59 Yatap-Ro, Bundang-Gu, Seongnam-Si, Gyeonggi-Do 463-712 Republic of Korea; 2 CHA University School of Medicine, Seongnam, Republic of Korea

**Keywords:** Neuroscience, Psychology

## Abstract

Self-forgiveness (SF) involves a process through which negative moral emotions directed at the self are replaced by benevolence and acceptance. Lower SF scores can be associated with less self-compassion, higher psychological distress, and lower life dissatisfaction. However, neural correlates of SF have not been investigated yet. We enrolled a total of 79 healthy individuals. The Self-Forgiveness Scale (SFS), Self-Compassion Scale (SCS), Connor-Davidson Resilience Scale (CD-RISC), Beck Depression Inventory-II (BDI-II), and Beck Anxiety Inventory (BAI) were evaluated. Voxel-wise correlational analyses showed a significant positive correlation between the total SFS scores and gray matter volumes (GMVs) in the fusiform gyrus (FG). In addition, the GMVs in the FG were significantly positively associated with the total SCS and CD-RISC scores and negatively correlated with the total BDI-II and BAI scores. These findings suggest that the FG related to the mirror neuron system might be a neural correlate of SF. Furthermore, its increased volumes of FG in healthy individuals can be associated with the capacity to overcome stressful life events.

## Introduction

Self-forgiveness (SF) involves a process through which negative emotions directed at the self (such as shame, guilt, or anger) are replaced with benevolence and acceptance^1^. In particular, some researchers have suggested that SF can be highly related to an individual’s personality traits rather than states that change according to the environment^2,3^. SF can be a supplementary technique in loving-kindness meditation^4^. The SF can be associated with self-compassion and psychological resilience^5,6^. Also, high SF is associated with a decrease in negative cognition, such as self-blame appraisal, a reduction of negative emotions, or a decrease in life dissatisfaction^7–9^.

To understand the connection between SF and the psychological characteristics described^10^, a transactional theory—developed by Lazarus and Folkman—on the relationship between psychological stress and coping can be applied. According to this theory, SF can be categorized as an adaptive emotion-focused coping response to the stress of self-condemnation (e.g., guilt, shame, and anger)^11^. Moreover, there is a possibility that SF can mediate or moderate the process of recovery to mental health, which can result in changes in self-compassion, resilience, depression, and anxiety^11^. Therefore, considering this stress-coping theory, SF—as an adaptive approach to stress—may be related to the psychological characteristics of an individual^12–14^.

In addition, a psychological model of SF showed that negative moral emotions such as shame, guilt, and self-blame play an essential role in the formation of SF^15^. In particular, SF may be associated with reducing or eliminating self-condemning emotions, such as shame or guilt. Hall et al.^16^ showed another psychological model of SF that explains that SF results from shame, guilt, attributions of responsibility, empathy, conciliatory behaviors, and perception of forgiveness from others. According to his model, guilt is initially positively correlated with SF and can facilitate SF^17,18^ as people forgive themselves, feelings of guilt decrease, and conciliatory behaviors tend to increase^19^. Additionally, SF and self-compassion appear to have much in common, mediated by reducing shame in healthy individuals^20^.

Considering that the SF is related to regulating moral emotions such as guilt or shame in the proposed SF model, it will be meaningful to find neuroanatomical neural correlates with guilt- or shame-related brain regions in healthy individuals. Guilt-related brain regions have been studied, including the fusiform gyrus (FG), middle temporal gyrus, amygdala, and insula^18,21^. The shame-related brain regions are the anterior cingulate cortex and parahippocampal gyrus in the temporal lobe, medial frontal gyrus, and inferior frontal gyrus have been studied^18^. These results suggest that shame and guilt have distinct activation regions and share some neural networks. Therefore, it may be inferred that the fronto-temporo-limbic regions play an important role in generating moral emotions.

Moreover, SF was associated with high emotional well-being or self-esteem. Those who are good at SF are more friendly and sympathetic than those who are not^7,22^. Furthermore, individuals with a high SF have healthy psychological characteristics. Another study found that people good at SF feel less shame^23^. Those who genuinely forgive themselves have lower levels of neuroticism^16^ and lower levels of psychological distress, such as anxiety and depression^7,22,24^. Therefore, it is essential to identify neural correlates associated with high SF that are correlated with changes in low levels of psychological distress, such as anxiety or depression. However, there are only a few studies on the direct structural neural correlations of SF among healthy individuals.

The present study investigated the structural neural correlates of SF in whole-brain regions of healthy individuals. It was hypothesized that (1) there would be a correlation between SF and gray matter volumes (GMVs) in moral emotions such as shame- or guilt-related brain regions, and (2) there might be a relationship between SF-related GMVs and psychological characteristics (self-compassion, psychological resilience, psychological-emotional distress, and life satisfaction) in healthy individuals. Therefore, we will perform whole-brain voxel-wise analyses to detect the neuroanatomical neural correlates of SF in healthy individuals and determine the relationship between psychological characteristics and SF-related brain regions.

## Methods

### Participants

A total of 79 (36 men and 43 women) healthy right-handed individuals were recruited from the local Korean community through advertisements. All participants were between 23 and 61 years old (mean 36.68 years), underwent magnetic resonance imaging (MRI), and completed a psychological assessment such as SF.

Healthy adults were excluded based on the following criteria: history of major psychiatric disorders, including schizophrenia spectrum disorders, mood disorders, anxiety disorders, and substance use disorders; neurological disorders and traumatic brain injury; pregnancy; and any prohibition of brain MRI scanning. Additionally, none of the participants had an individual or primary-relative family history of psychiatric disorders according to the review of the *Diagnostic and Statistical Manual of Mental Disorders*, *Fourth Edition*, *Text Revision* (*DSM-IV-TR)* screening by interview.

All procedures were performed after review and approval by the Institutional Review Board of the CHA Bundang Medical Center (2019-05-030). After the participants had a detailed explanation of the study, written informed consent was obtained from the latest version of the Declaration of Helsinki. Further, the principles of Good Clinical Practice were acquired.

### Dispositional self-forgiveness assessment

The Self-Forgiveness Scale (SFS) by Kim et al. measures dispositional SF as the process of responding by changing from a negative and punitive response to understanding and generosity about one’s actions that are inappropriate for one’s thoughts^25^. This scale consisted of 19 items used among healthy individuals, each rated on a 1- to 5-point scale, with the SFS score ranging from 19 to 95. The higher the total SFS scores, the greater the SF score in healthy individuals. Additionally, we used the three subscales of the SFS consisting of “acceptance and improvement,” “transferring of responsibility,” and “negative affect, thought, and behavior.” First, the “acceptance and improvement” subscale consists of accepting oneself to make mistakes and believing that one can do better in the future. Next, the “transferring of responsibility” subscale contains contents such as blaming others, disapproval, and avoidance and indicates a tendency to avoid responsibility for one’s wrongdoing and attribute it to others or circumstances. Additionally, the “negative affect, thought, and behavior” subscale refers to the tendency to be unable to escape from negative affect, thought, and behavior regarding one’s faults. Among the three subscales of SF, “transferring of responsibility” and “negative affect, thought, and behavior” have opposite meanings to SF. These two subscales were therefore counted as inverse scores when calculating the total SF scores, whereas they were counted as untransformed scores when performing the analysis for each subscale. Moreover, the SFS has a high Cronbach’s *α* coefficient of 0.87. To obtain the total SFS scores, the inversely transformed values of “transferring responsibility” and “negative affect, thought, and behavior” were used.

### Other psychological characteristics assessments

To evaluate the levels of self-compassion, we used the Korean version of the Self-Compassion Scale (SCS) by Neff and Kim et al., a self-reported test consisting of a 5-point scale with high internal consistency (Cronbach’s *α* = 0.90) for the total scores. Further, it ranges between 0.74 and 0.81 for the six subscale scores^26,27^. It consists of 26 items, including five items for self-kindness, five items for self-judgment, and four items for common humanity, isolation, mindfulness, and over-identification. There were 13 reverse-scoring items out of the total items, and the total scores for self-compassion were calculated by adding up the individual subscale scores^27^. The scores for the negative subscales (i.e., self-judgment, isolation, and over-identification) were reverse-coded, and then the SCS total scores were calculated by averaging the six subscale means.

The Korean version of the Connor-Davidson Resilience Scale (CD-RISC), which contains 25 items, was used to evaluate resilience as the ability to cope with stress and adversity^28^. Connor and Davidson (2003) focused on the CD-RISC as a modifiable trait, and the CD-RISC contains items for evaluating resilience with a high internal consistency (Cronbach’s *α* = 0.93).

Additionally, the Korean versions of the Beck Depression Inventory-II (BDI-II) and Beck Anxiety Inventory (BAI) were used to evaluate psychological distress, such as the severity of depression and anxiety. The BDI-II consists of 21 self-administered items, ranging from 0 to 63^29^. The BAI has 21 self-reported inventories for assessing clinical anxiety severity on a scale of 0 to 3^29^.

### Image acquisition

A total of 79 participants underwent brain MRI on a GE Signa HDxt 3.0-Tesla scanner (GE Healthcare, Milwaukee, WI, USA) with an eight-channel phased-array head coil. Three dimensional T1-weighted fast spoiled gradient recalled echo sequence has the parameters that repetition time = 6.3 ms, echo time = 2.1 ms, flip angle = 12º, slice thickness = 1 mm, field of view = 256 × 256 mm^2^, matrix = 256 × 256, and isotropic voxel size = 1 × 1 × 1 mm^3^.

### Statistical analyses

For analyzing GMVs, voxel-based analysis was performed using FreeSurfer (version 7.1.0; http://surfer.nmr.mgh.harvard.edu) with standard images processed using the standard form in FreeSurfer. In addition, the Destrieux atlas was used as a reference to label and calculate the mean value of GMVs, which was implemented in FreeSurfer^30,31^.

Multiple regression models with age, sex, year of education, and total intracranial volume (ICV) as covariates were performed in a healthy adult database. The results were corrected for multiple comparisons using a Monte Carlo simulation with a *p*-value of < 0.05.

And, the objectives of our study were to evaluate the correlation of GMVs in the neural correlates of SF with psychological characteristics and to determine the implications of these regions. Therefore, exploratory Pearson’s correlation analyses were conducted between the GMVs of the neural correlates of SF and the scores of the psychological measurements (SCS, CD-RISC, BDI-II, and BAI). For analyses, possible outlier values were examined using adjusted standardized sores.

Except for voxel-wise analysis, statistical analyses were performed using the Statistical Package for the Social Sciences (SPSS) Windows software version 27. A statistical *p*-value less than 0.05 was considered significant.

## Results

### Sociodemographic and clinical characteristics

Table 1 shows the sociodemographic and clinical characteristics of a total of 79 participants. Furthermore, there were no differences in SFS scores according to sociodemographic variables, such as age, sex, education level, presence of religion, marital status, and monthly income. Additionally, statistical non-significance was maintained when adjusting for covariates (age, sex, and ICV).

**Table 1:** Sociodemographic and clinical characteristics in healthy individuals.

Sociodemographics (total *N* = 79)	*N* (%) or mean (± SD)	Range (min–max)
Sex: men/women	36 (45.60) /43 (54.40)	
Age (years)	36.68 (± 8.33)	23.00–61.00
Education year (years)	17.56 (± 2.14)	12.00–23.00
Intracranial volume (mL)	1537.96 (± 127.08)	1255.00–1834.00
Religion: existed/none	37 (61.67) /23 (38.33)	
Marital status: living with partner/without partner	34 (50.00) /34 (50.00)	
Monthly income: ≥ 1800 $USD/< 1800 $USD	57 (95.00)/3 (5.00)	
Clinical characteristics (total *N* = 79)	*N* (%) or mean (± SD)	Range (min–max)
Self-forgiveness (SFS) total scores	72.60 (± 8.30)	54.00–92.00
Acceptance and improvement	30.86 (± 4.62)	19.00–40.00
Transferring of responsibility	19.39 (± 2.61)	11.00–25.00
Negative affect, thought and behavior	22.19 (± 4.24)	12.00–30.00
Self-compassion (SCS) total scores	90.95 (± 10.58)	65.00–119.00
Self-kindness	13.40 (± 3.69)	5.00–21.00
Self-judgement	21.03 (± 3.30)	12.00–25.00
Common humanity	11.39 (± 3.55)	4.00–20.00
Isolation	17.50 (± 2.36)	11.00–20.00
Mindfulness	12.50 (± 3.00)	7.00–19.00
Over identification	15.64 (± 2.80)	7.00–20.00
Resilience (CD-RISC) total scores	62.97 (± 9.35)	46.00–89.00
Depression (BDI-II) total scores	4.63 (± 4.92)	0.00–21.00
Anxiety (BAI) total scores	2.92 (± 3.80)	0.00–18.00

Values represent count (percent), *SD* Standard Deviation, *N* Subject Number, *SFS* Self-Forgiveness scale, *SCS* Self-Compassion Scale, *CD-RISC* Connor and Davidson Resilience Scale, *BDI-II* Beck Depression Inventory-II, *BAI* Beck Anxiety Inventory.

### Voxel-wise correlation in whole-brain analyses between the self-forgiveness and the gray matter volume in healthy individuals

**Figure 1:**
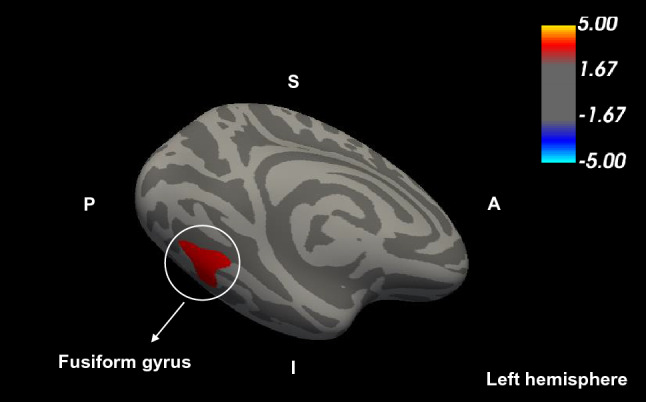
Result of the whole brain voxel-wise correlational analysis. The regions including fusiform gyrus [Montreal Neurologic Institute (MNI) coordinate *X* = − 29.4, *Y* = − 70.9, *Z* = − 13.3] indicating significantly positively correlated gray matter volumes for the high Self-Forgiveness Scale total scores in healthy individuals are shown in red (Monte Carlo simulations correction, cluster-wise *p* < 0.05). Images overlaid on the MNI 1 mm template. The color scale bar shows the logarithmic scale of *p*-values (− log10).

The total SFS scores were significantly positively correlated with the GMVs in the left FG (peak voxel of MNI: *X* =  − 29.4, *Y* =  − 70.9, *Z* =  − 13.3, Monte Carlo simulation correction, cluster-wise *p* < 0.05) in healthy individuals (Fig. 1). Additionally, statistical significance was maintained when adjusting for covariates, such as age, sex, and ICV.

### Voxel-wise correlation in whole-brain analyses between the three subscales of self-forgiveness scale and the gray matter volume in healthy individuals

The total scores of each subscale of the SFS showed a significant correlation with GMVs in several brain regions. Our study showed a significant positive correlation between the subscale scores of ‘acceptance and improvement’ subscale and GMVs of the left cuneus cortex (peak voxel of MNI: *X* = − 5.6, *Y* = − 78.3, *Z* = − 24.0, Monte Carlo simulations correction, cluster-wise *p* < 0.05) and right posterior parietal cortex (PPC) (peak voxel of MNI: *X* = 12.7, *Y* = − 90.5, *Z* = 20.5, Monte Carlo simulations correction, cluster-wise *p* < 0.05) in healthy individuals. Furthermore, the scores of “transferring of responsibility” subscale were positively correlated with GMVs of the left precuneus cortex (peak voxel of MNI: *X* = − 20.1, *Y* = − 61.7, *Z* = 10.8, Monte Carlo simulations correction, cluster-wise *p* < 0.05) and the right parahippocampal gyrus (peak voxel of MNI: *X* = 17.0, *Y* = − 38.2, *Z* = − 6.6, Monte Carlo simulations correction, cluster-wise *p* < 0.05) in healthy individuals. However, the “negative affect, thought, and behavior” subscales comprised inverse questions. That is, the lower the degree of “negative emotion, thought, and behavior,” the higher the degree of SF. In this study, the lower the subscale score, the significantly higher the GMVs of FG (peak voxel of MNI: X = − 29.7, Y = − 70.3, Z = − 12.5, Monte Carlo simulations correction, cluster-wise *p* < 0.05) in healthy individuals.

### Exploratory correlation analyses between the gray matter volumes of the fusiform gyrus associated with the self-forgiveness scale’s total scores and psychological characteristics in healthy individuals

**Figure 2:**
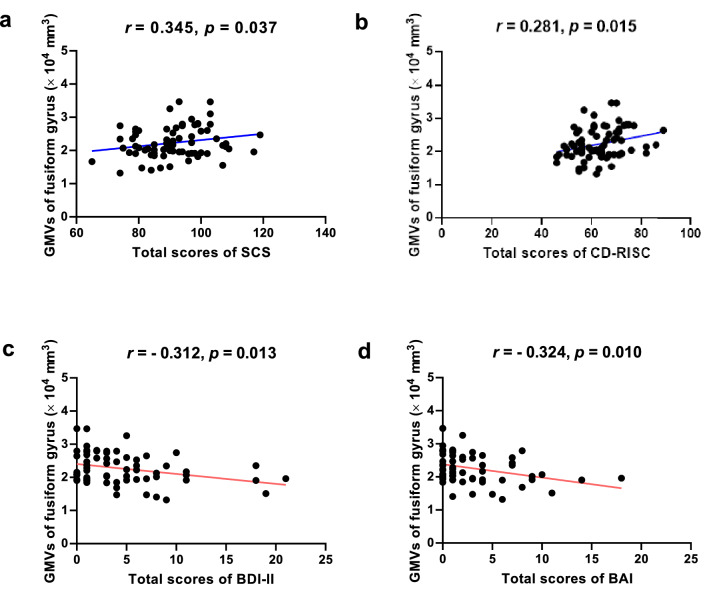
Exploratory correlation analyses revealed the significant associations between the total scores of psychological characteristics [(**a**) self-compassion (SCS), (**b**) resilience (CD-RISC), (**c**) depression (BDI-II), and (**d**) anxiety (BAI)]. Further, GMVs in the fusiform gyrus are significantly associated with the total SFS scores in healthy individuals. *CD-RISC* Connor-Davidson Resilience Scale, *SCS* Self-Compassion Scale, *BDI-II* Beck Depression Inventory-II, *BAI* Beck Anxiety Inventory, *SFS* Self-Forgiveness Scale, *GMVs* gray matter volumes.

Our study found one potential outlier, which was subsequently excluded from our analyses. Thereafter, the exploratory correlation analyses showed more significant *p*-values than before in the CD-RISC, BDI-II, and BAI scales. There are significant positive correlations between the GMVs in the left FG and the total scores of the SCS and CD-RISC in healthy individuals. However, the higher the GMVs in the left FG, the lower the total BDI-II and BAI scores in healthy individuals (Fig. 2).

### Exploratory correlation analyses between the gray matter volumes of self-forgiveness-related regions and psychological characteristics in healthy individuals

**Figure 3:**
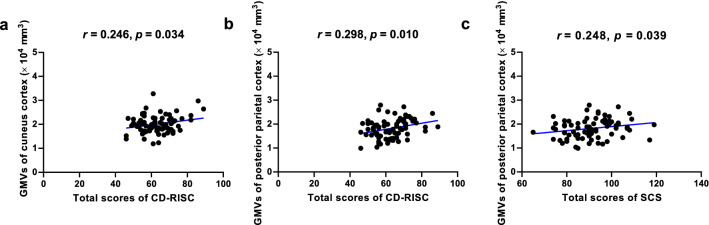
Exploratory correlational analyses showed the significant associations between GMVs in the cuneus cortex and posterior parietal cortex related to the subscale (acceptance and improvement) of Self-Forgiveness Scale total scores and the total scores of psychological characteristics [(**a**) and (**b**) resilience (CD-RISC), and (**c**) self-compassion (SCS)] among healthy individuals. *GMVs* Gray matter volumes, *CD-RISC* Connor-Davidson Resilience Scale, *SCS* Self-Compassion Scale.

Figure 3 showed that the GMVs in the left cuneus cortex, including “acceptance and improvement” subscale-related brain regions, were significantly positively associated with healthy individuals’ total CD-RISC scores. The GMVs of the right PPC showed a significant positive correlation with the total CD-RISC and SCS scores in healthy individuals (all *p*-value < 0.05). Further, after excluding one potential outlier, the levels of significance were stronger than those before in the CD-RISC scale-related correlation analyses (Fig. 3).

## Discussion

Our study is the first to demonstrate that a high SF was significantly correlated with greater GMVs in the FG, which was positively correlated with self-compassion and resilience and negatively associated with psychological distress such as depression and anxiety among healthy individuals. Additionally, concerning subscale analyses, the changes in GMVs in the cuneus cortex, PPC, and FG related to SF may be associated with self-compassion and resilience among healthy individuals.

This study showed that the higher the level of SF, the higher the GMVs in the FG among healthy individuals. According to the SF model, SF could relate to empathy and critical emotions, such as shame and guilt^32^. A previous study on multidimensional empathy revealed that individuals who could take others’ perspectives were prone to have higher SF^33^. Another study showed that lower empathy could be associated with reduced activation of the FG in autism spectrum disorder^34^.

There are significant positive correlations between the level of guilt and facial recognition^35^. Among the vital neural regions related to facial emotional recognition of themselves or others, the FG is one of the core neural regions that is activated for the forgiveness of others or guilt states^17,18,21,36–39^. Guilt is initially thought to be positively correlated with SF^40,41^. Therefore, our finding—that SF may be associated with FG—is consistent with previous findings.

Our research indicated that greater GMVs in the FG related to high SF levels were associated with higher self-compassion and resilience and less severe psychological distress such as depression or anxiety. Generally, both SF and self-compassion appear to have similarities in that they relate to emotional regulation, use adaptive means of self-regulation, and affect broad psychological functions^20^. Furthermore, previous studies show that SF associated with self-compassion support this finding^5,42^.

Regarding the relationship between SF and resilience, one study^6^ showed that SF could increase resilience and reduce suicidal ideation in older adults. Further, a structural neuroimaging study indicated that increased cortical thickness in the FG is observed in highly resilient individuals^43^.

The SF has been presented to be negatively associated with the levels of depression and anxiety^44^. According to a systematic review, self-blame, which can be considered the opposite of SF, may predict depression, anxiety, and posttraumatic symptomology in bereaved parents. Our finding of this negative correlation could result from the potential role of low self-blame.

Our study showed that the higher the “acceptance and improvement” subscale scores, the higher the GMVs in the cuneus cortex and PPC, which are related to emotional attention and emotional regulation^45,46^. The subscale “negative affect, thought, and behavior” compromise inverse questions and is negatively correlated with the GMVs in the FG. That is, higher the negative affect, thought, and behavior can be related to lower GMVs of FG among healthy individuals. Taken together, it can be assumed that the GMVs of these brain regions are related to emotional regulation or recognition associated with SF in healthy individuals.

Our findings showed that healthy individuals with less transfer of responsibility had smaller GMVs in the precuneus and parahippocampal gyri. In other words, healthy individuals who blame themselves may have a smaller GMVs in the precuneus and parahippocampal gyrus. The precuneus is part of the DMN and is involved in self-referential processing^47–49^. The parahippocampal gyrus is involved in the retrieval of negative contextual memory^50^. Therefore, these findings suggest that individuals who blame themselves are more likely to have a smaller GMVs in the precuneus and parahippocampal gyrus.

Meanwhile, a mirror neuron system (MNS) is a group of neurons mirroring the behavior of others, and plays an essential role in imitation and empathy^51^. The three major components of the human classical MNS include the superior temporal sulcus (STS), inferior parietal lobule, and complex inferior frontal gyrus and premotor cortex^52–54^. Empathy is the tendency of observers to project themselves into their observations, a process referred to as an inner imitation^55^. Mirroring of emotional facial expression appears to be involved in not only the above-mentioned classical MNS regions but also extended MNS regions, including the face processing networks of the fusiform gyrus^56,57^. Moreover, previous research has presented that SF and empathy are likely related to each other^58,59^, which suggests that SF may be related to MNS. Structural changes in MNS-related brain regions may be associated with the capacity to empathize, self-compassion, and low psychological distress among healthy individuals.

Our study had several limitations. First, we only investigated the association between brain structure and SF using a relatively small sample size (*n* = 79). Therefore, it may be necessary to examine the neurostructural correlates of SF in larger participant numbers. Second, the present study focused on the exploratory correlation between brain structures and psychological characteristics. Thus, further validation is required to establish causal relationships.

In conclusion, our study suggested that healthy individuals with high SF showed increased GMVs in the FG, presumably mediated by guilt or shame. Their correlations were associated with other psychological characteristics (high resilience, high self-compassion, high life satisfaction, and low psychological distress). Moreover, in the subscale analyses, the GMVs in some brain regions (cuneus cortex, PPC, precuneus cortex, parahippocampal gyrus, and FG) showed a significant positive correlation with self-compassion and psychological resilience. Further, they showed life satisfaction and a negative association with psychological distress such as depression and anxiety in healthy individuals. This result implies that SF is related to FG, which is responsible for facial recognition and emotional regulation and can be included in the mirror neuron system. These findings may provide a neuroscientific basis for understanding SF.

## Data Availability

All data used or analyzed during this study were included in this article. And further request can be directed with the detailed description to the corresponding authors.
