# Author Correction: Self-forgiveness is associated with increased volumes of fusiform gyrus in healthy individuals

**DOI:** 10.1038/s41598-023-34497-x

**Published:** 2023-05-04

**Authors:** Hyun-Ju Kim, Junghwa Seo, Minji Bang, Sang-Hyuk Lee

**Affiliations:** 1 Department of Psychiatry, CHA Bundang Medical Center, CHA University School of Medicine, 59 Yatap-Ro, Bundang-Gu, Seongnam-Si, Gyeonggi-Do 463-712 Republic of Korea; 2 CHA University School of Medicine, Seongnam, Republic of Korea

Correction to: *Scientific Reports* 10.1038/s41598-023-32731-0, published online 04 April 2023

In the original version of this Article, Hyun‑Ju Kim, Junghwa Seo and Minji Bang were incorrectly listed as corresponding authors. The correct corresponding author for this Article is Sang-Hyuk Lee. Correspondence and request for materials should be addressed to drshlee@cha.ac.kr.

The original Article has been corrected.

